# Microglia sensing of peripheral signals that bridge the brain and body

**DOI:** 10.1186/s13024-025-00905-1

**Published:** 2025-10-29

**Authors:** Claire E. Young, Melanie A. Samuel

**Affiliations:** 1 Department of Neuroscience, Baylor College of Medicine, Houston, TX 77030 USA; 2 Jan and Dan Duncan Neurological Research Institute at Texas Children’s Hospital, Houston, TX 77030 USA; 3 Huffington Center on Aging, Baylor College of Medicine, Houston, TX 77030 USA

**Keywords:** Microglia, Central nervous system, Peripheral cues, Neurodegenerative disease, Neurons

## Abstract

Microglia are the resident immune cell of the brain, and alterations in microglia signaling have been implicated in many neurodegenerative disorders. While microglia responses to central cues and other brain cell types are well documented, studies are increasingly investigating the impact of peripherally derived signals on microglia function. A diverse array of peripheral cues, including dietary components, hormones, and bacteria metabolites and components from the microbiome cross the blood brain barrier and directly influence microglia state through ligand-receptor interactions. This review highlights the complexity of brain-body interactions from the perspective of microglia function and proposes the idea that microglia could serve as a central hub of detection and regulation of body state changes. In addition, improving understanding of how microglia respond to peripheral cues will allow for improved preclinical experimental design. As peripheral cues have the potential to be more readily manipulated than central cues, these interactions also have implications for the treatment of many diseases and neurodegenerative disorders.

**Graphical Abstract:**

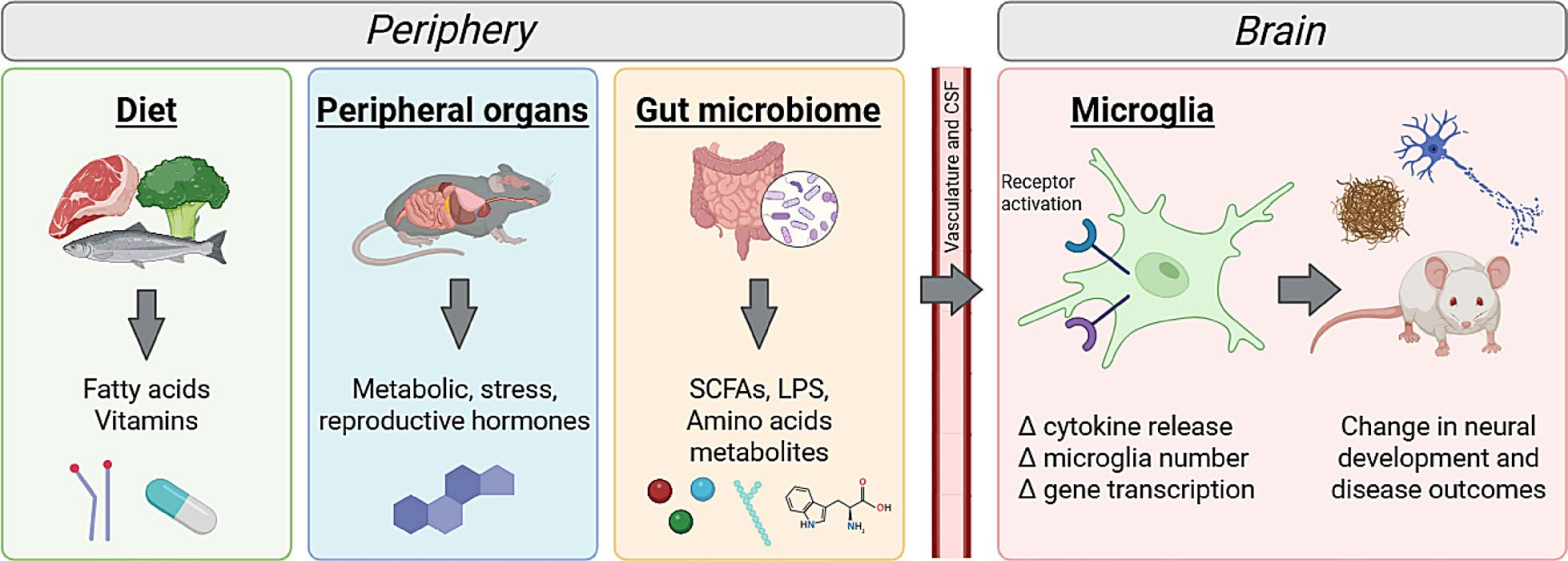

## Introduction

**Fig. 1:**
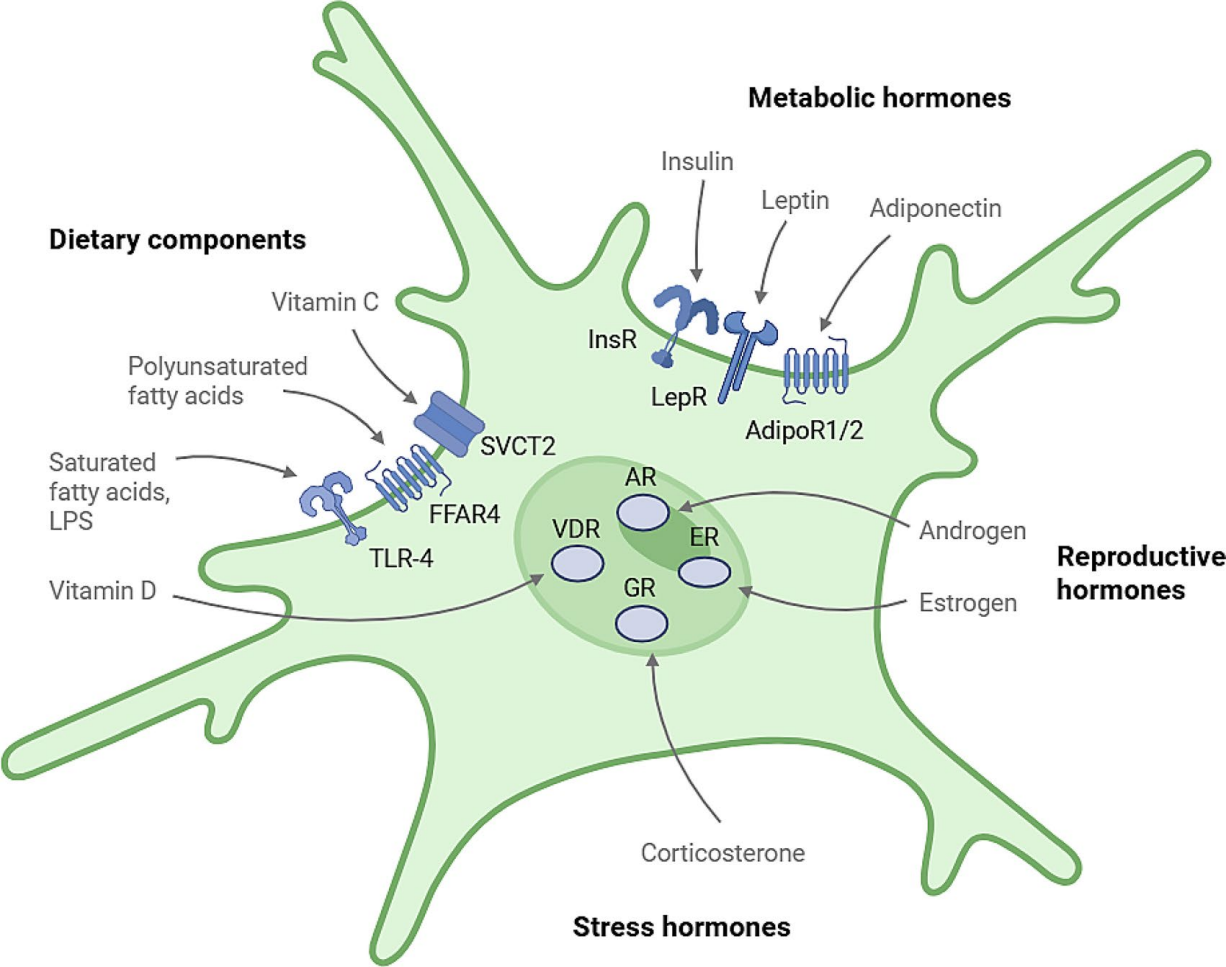
Microglia expression of receptors to peripheral cues. Microglia expression of receptors to different peripheral cues allows for direct interactions between the periphery and microglia state. Receptor activation causes changes in intracellular signaling pathways that results in changes in microglia reactivity, including cytokine release, morphology, and phagocytic state depending on the cue. Microglia receptors are an active area of research, and likely more will be identified in the future. Studies looking at microglia-specific knockouts of these receptors will help validate their importance for microglia function

As the tissue-resident macrophage in the central nervous system (CNS), microglia respond to local injury and infection to maintain homeostasis. Since their discovery over 100 years ago, microglia have been recognized for their many roles, including synapse pruning during development, modulation of the CNS inflammatory response, and dynamic surveillance of their environment (reviewed in Nayak, Roth et al. [1], Borst, Dumas et al. [2]. The regulation of microglia state is a highly dynamic process and has been implicated in the progression of neurodegenerative disorders, as reviewed in Gao, Jiang et al. [3]. While microglia are traditionally studied for their role in CNS health through their recognition and response to signals produced by the CNS in development and disease [4], microglia can also respond to many peripherally derived dietary compounds, hormones, and bacteria components through a diverse set of receptors and transporters (Fig. 1). This raises the interesting possibility that microglia can respond to and may help regulate whole-body homeostasis. Peripheral compounds can enter the extracellular space in the brain through crossing the blood brain barrier (BBB) or through the cerebral spinal fluid (CSF) via diffusion from capillaries into the choroid plexus [5]. Additionally, microglia in circumventricular organs may have greater contact with peripheral cues. These are brain regions around the third and fourth ventricle with a semi-permeable BBB that allows for more molecules to cross [6].

In this review, we propose the idea that microglia could serve as a key integrative hub for sensing and responding to peripheral cues. To illustrate this idea, we focus here on three types of peripheral cues. We begin by discussing the types of dietary components that microglia can sense and delineate how these diverse responses impact microglia function. We then outline the peripherally derived hormones to which microglia are responsive and explore how hormonal regulation of microglia may impact brain and body outcomes. We next address the bacterial metabolites that can be sensed by microglia and detail how these signals can impact microglia function. In each case, we outline how microglia peripheral cue sensing may differ in normal adulthood and in disease. We prioritize these cues both because microglia express appropriate receptors for these signals and because several of these cues have been studied with microglia-specific receptor manipulations to demonstrate a direct effect on microglia signaling. We suggest that these studies help provide a framework and rationale for additional future investigation of microglia as an important sensor and regulator of body state changes. We also highlight the possibility for designing minimally invasive treatments that could target microglia reactivity and function across disease states, including neurodegenerative disorders.

## Dietary components

Dietary components, such as dietary fats and vitamins, can directly alter microglia activity. The response of microglia to dietary components has been primarily studied in the context of high fat diet (HFD) feeding. Broadly speaking, both acute and chronic HFD feeding has been found to increase microglia reactivity in the arcuate nucleus, a hypothalamic hub for neural feeding regulation [7, 8]. How other dietary components cause changes in microglia signaling is an emerging field of research and is detailed below. Understanding how diet impacts microglia function is a question of translational significance, as diet is known to influence risk for conditions such as obesity, diabetes, and cardiovascular disease [9]. Particularly in the context of neurodegenerative disease, dietary interventions such as the Mediterranean-DASH Intervention for Neurodegenerative Delay (MIND) diet have been shown to reduce Alzheimer’s disease (AD) risk [10]. Thus, understanding how diet influences microglia activity may help inform future diet and lifestyle interventions. We discuss known receptors and pathways through which microglia respond to fatty acids and vitamins and highlight critical knowledge gaps.

### Fatty acids

Dietary fatty acids can exert either pro- or anti-inflammatory effects on microglia depending on the fatty acid type. Dietary fatty acids can be broadly divided into two groups: saturated fatty acids (SFAs), which are solid at room temperature, and unsaturated or polyunsaturated fatty acids (PUFAs), which are liquid at room temperature. Dietary fatty acids can cross the BBB [11], as seen with changes in brain fatty acid content following HFD feeding [8], and can act as signaling molecules to microglia through their action on fat receptors. These include free fatty acid receptor 4 (FFAR4; also known as GPR120) which binds to long chain fatty acids, particularly PUFAs [12, 13] and TLR4, which binds to many pathogen-associated molecular patterns and SFAs [14, 15]. When bound by long chain fatty acids, FFAR4 mediates an anti-inflammatory effect in microglia through β-arrestin-2 recruitment, which downregulates NF-κB signaling [13, 16]. β-arrestin-2 recruitment is thought to induce anti-inflammatory responses by interacting with TAB1, which inhibits TAK1 activity and subsequent NF-κB activation [13]. In line with this, microglia-specific FFAR4 knockout resulted in more reactive microglia morphology and increased expression of phagocytosis and inflammatory markers in HFD-fed adult mice [16].

In contrast, SFA treatment with palmitic acid has been shown to increase pro-inflammatory cytokine production, including TNF-α, IL-1β, and IL-6, in the immortalized BV-2 microglia-like cell line through increasing NF-κB signaling in a TLR4 dependent manner [15]. TLR4 activation increases TAK1 activity, which promotes NF-κB activation [14]. These data indicate the importance of the NF-κB response to diet on microglia state and highlight the varying effects of different fat receptor activation. This is recapitulated in vivo, where SFA feeding was found to be pro-inflammatory in the hypothalamus, primarily through TLR4 receptors on microglia [17]. The effect of SFAs on microglia in vivo is not solely due to weight gain caused by HFD feeding, as oral gavage of isolated SFAs also increased hypothalamic cytokine expression [8]. Together with previous studies, this work suggests that SFA sensing by microglia can directly promote microglia cytokine production by binding to and activating TLR4. However, some evidence suggests that SFAs do not act as a direct TLR4 ligand and instead induce other cellular changes, such as alterations to lipid metabolic pathways, membrane lipid composition, and cellular metabolism, that promote a pro-inflammatory response [18]. Since much of the work on fatty acid induced responses in microglia has been done in vitro, future studies testing the effect of different fatty acid compositions on microglia reactivity in vivo are needed, particularly with microglia-specific receptor knock out animals to help elucidate signaling pathways involved in fatty acid responses.

**Fig. 2:**
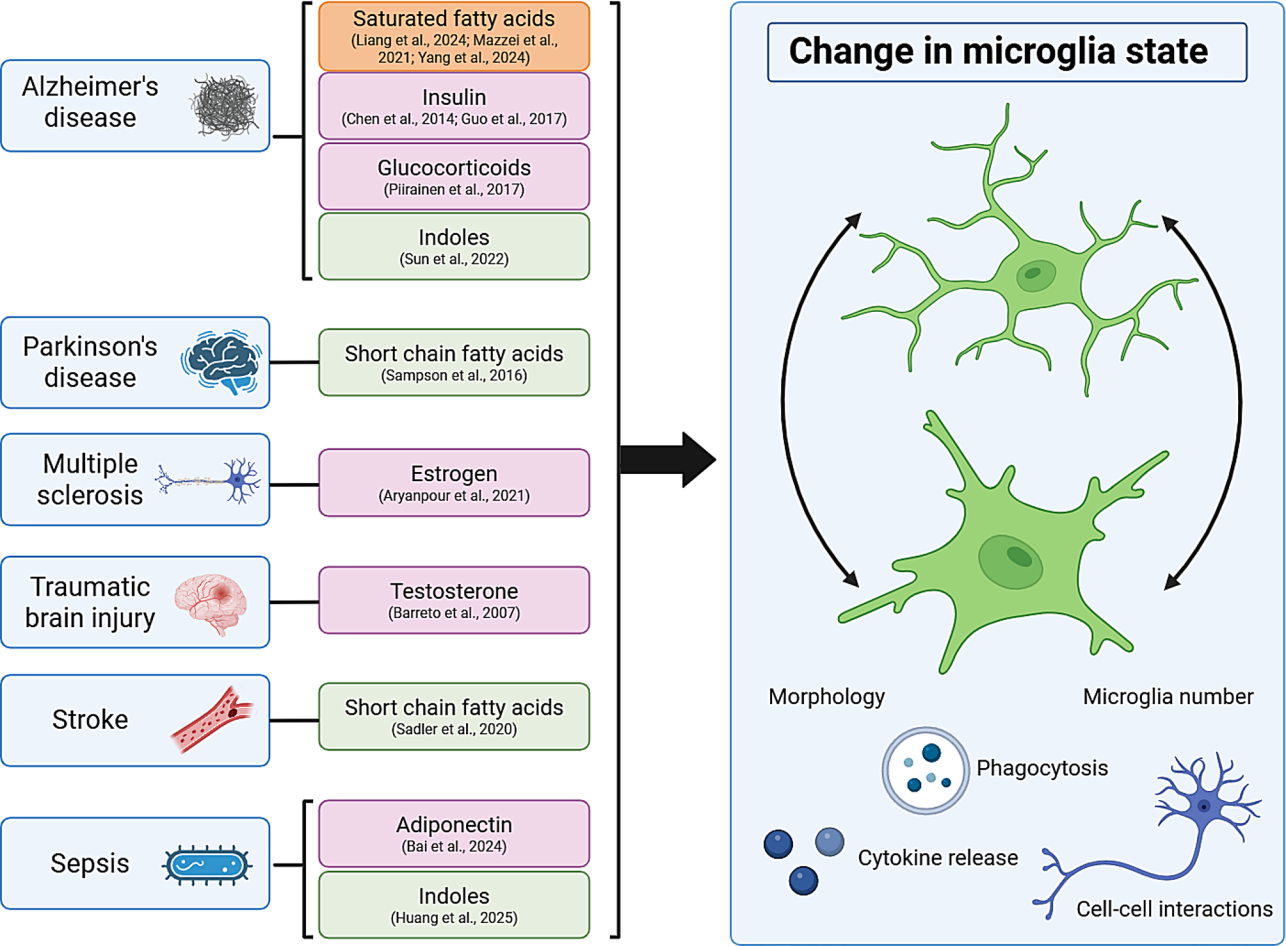
Peripheral cues implicated in disease progression through modulation of microglia signaling. Many peripheral cues have been studied for potential therapeutic benefit in disease contexts by their modulation of microglia state. In the figure, peripheral cues are listed next to a disease in the context of which they have been shown to influence microglia outcomes. Cues related to dietary compounds are listed in orange, hormones in pink, and gut microbiome components in green. These cues can alter microglia state and may affect disease outcomes by altering microglia morphology, number, phagocytosis, cytokine release, and interactions with other cell types such as neurons. Because these cues can be administered in a less invasive manner than direct brain manipulations, they pose a translationally relevant area of investigation. It will be important for future studies of peripheral cues to examine if changes in microglia properties, such as morphology or phagocytosis, have a physiologically relevant effect on disease outcomes. Beyond therapeutic use, it is also important to study how basal levels of these cues affect disease development and progression

The effect of dietary fatty acids on microglia in the context of aging and AD have been extensively studied, although most of the studies are correlative in nature. The link between different peripheral cues and disease states are outlined in Fig. 2. In aged mice, chronic HFD feeding was found to increase microglia reactivity in the hippocampus [19]. Likewise, a study looking at 1-year HFD feeding found an increase in microglia reactivity in the hippocampus that was ameliorated by TREM2 overexpression [20]. In multiple mouse models of AD, including 3xTg-AD [21], *App*^*NL−F/NL−F*^ [22], and APP/PS1 [23] mice, chronic HFD feeding was found to increase microglia reactivity in the hippocampus and impair microglia β-amyloid (Aβ) plaque clearance. The type and duration of HFD feeding differed between all these studies, which is a significant source of variation in AD model research that complicates study interpretation. For example, dietary feeding duration can range from 2 months to over a year and fat percentages can range from 40 to 60% kcal from fat with or without added cholesterol [24]. Despite the many studies that observe an increase in microglia reactivity and AD pathology with HFD feeding, there is a lack of mechanistic evidence as to what is driving these changes. For example, it is unclear to what degree microglia respond directly versus indirectly to HFD-induced changes. For the latter, it is possible that other aging or AD-related changes, such as circadian disruption, changes in the stress response, or microbiome alterations, are underlying the changes in microglia function. Studying microglia-specific receptor knock out models in aged mice and in mouse AD models would help determine causality.

### Vitamins

Micronutrient availability has been shown to directly influence the microglia inflammatory response through signaling via vitamin receptors and transporters on microglia. Among these is vitamin D and its receptor, vitamin D receptor (VDR). Vitamin D is obtained through diet or sun exposure and is then converted into the active form, called calcitriol, by the kidneys. Vitamin D has anti-inflammatory effects in disease models. In primary mouse microglia cell culture, calcitriol administration to microglia stimulated with IFNγ prevented a pro-inflammatory response in an IL-10/SOCS3 dependent manner. IL-10 was shown to induce the expression of SOCS3 (suppressor of cytokine signaling 3), which in turn bound Janus kinases and prevented their interaction with STAT proteins [25]. In a rat model of hypertension, calcitriol administration was found to reduce microglia Iba1 expression and expression of pro-inflammatory markers [26]. Conversely, myeloid-specific knockout of the vitamin D receptor enhances the microglia inflammatory response after cerebral ischemia [27]. Additional studies examining the effect of microglia VDR signaling in non-disease contexts will be important to establish how vitamin D contributes to microglia signaling under homeostatic conditions. Given that vitamin D deficiency is common in the United States, at around 41% prevalence in adults [28], further understanding of vitamin D actions on microglia is critical. Additionally, around 70% of children and adolescents in the United States were found to have deficient or insufficient vitamin D levels, raising the possibility of developmental alterations in microglia function [29].

Another vitamin with potential direct effects on microglia state is vitamin C (also known as ascorbic acid), which is transported into microglia through the sodium-dependent vitamin C transporter 2 (SVCT2) [30]. Like vitamin D, vitamin C has anti-inflammatory effects on microglia signaling and may also regulate microglia density. In a primary rat microglia cell culture model, knockdown of SVCT2 increased production of pro-inflammatory cytokines [31]. Mice heterozygous for SVCT2 also had increased microglia density with less ramified morphology [31]. Rats unable to synthesize vitamin C due to a mutation in the synthesis enzyme showed increased IL-6 expression in the hippocampus and more reactive microglia morphology [32]. While these results suggest a role for vitamin C signaling in microglia response, follow up studies using in vivo microglia-specific SVCT2 manipulation will help clarify if the anti-inflammatory effect is due to direct vitamin C action on microglia or wider systemic changes. Unlike humans who must obtain vitamin C from their diet, rats and mice are both able to synthesize vitamin C endogenously [33]. Thus, further research is needed to determine if vitamin C signaling plays an even larger role in human microglia state regulation considering that vitamin C is an essential nutrient.

Studies examining the impacts of other vitamins on microglia state have been largely performed in cell culture and mostly lack mechanistic explanations for how these vitamins modulate microglia signaling. One such vitamin is vitamin E (α-tocopherol), which acts as an antioxidant and signaling molecule. Vitamin E pre-treatment in N9 or BV2 microglia cell lines attenuated lipopolysaccharide (LPS)-induced inflammatory responses [34, 35]. While vitamin E is able to cross the BBB, its transport within the CNS has not been well studied. However, recent results suggest the potential for vitamin E transfer between astrocytes and neurons [36]. Understanding how vitamin E modulates microglia responses and communication between neurons and astrocytes are important future directions. Another vitamin studied in microglia signaling is vitamin K. Vitamin K is known best for its role in blood clotting but has recently been implicated in inflammatory regulation. While there are several forms of vitamin K, including K_1_ and K_2_, microglia studies to date have only investigated the impact of K_2_. K_2_ pre-treatment was found to attenuate LPS or rotenone-induced cytokine release and NF-κB activation in MG6 or BV2 microglia cells [37; 38]. In vivo, a diet low in vitamin K was found to reduce vitamin K concentration in the brain and reduce hippocampal microglia complexity [39]. Finally, a few studies have looked at the impact of vitamin A (retinol or retinoic acid) on microglia state in BV2 and primary rat cell culture and found that vitamin A mitigates LPS-induced inflammation via activation of the retinoic acid receptor [40, 41].

Given the direct impact of diet on microglia inflammatory response, more research into dietary factors influencing microglia state is needed to help inform dietary recommendations for brain health and to better understand the influence of diet on chronic diseases like obesity and AD. While outright vitamin deficiencies are rare in the United States, subclinical inadequacies in vitamin intake are common and implicated in neurodegenerative disorders such as AD [42, 43]. Thus testing the effect of mild vitamin deficiencies on microglia state in mice in both healthy and disease states will be important for maximizing translational potential. Finally, it is important not to assume that higher vitamin consumption is always beneficial for microglia function, as excessive vitamin consumption through supplementation, can cause toxicity [44, 45]. Because diet is multifaceted and has close links with hormonal responses (discussed in the next section), isolating the effects of single nutrients in vivo will require careful experiment planning.

## Hormones

Hormones are chemical messengers released from one organ that induce changes in target tissues. Given that microglia express receptors for a number of hormones (Table 1) and that peripheral hormones can cross the BBB through transporters or diffusion [57], hormone signaling allows for organs to directly influence microglia state and function. Three types of hormone signaling have been shown to directly influence microglia function—metabolic-related hormones, psychological stress hormones, and reproductive fitness hormones. We discuss each of these in turn and detail their influence on microglia state and function.

**Table 1:** Hormone receptors expressed by microglia

Ligand	Receptor	Activation effect on microglia	Citation
Insulin	InsR	Reduced cytokine release; less reactive morphology	[46, 47]
Leptin	LepR	Increased cytokine release	[48]
Adiponectin	AdipoR1/AdipoR2	Decreased cytokine release, increased quiescence	[49, 50]
Glucocorticoids/corticosterone	GR	Basal activation: anti-inflammatory response Chronic: pro-inflammatory response	[51,52]
Estrogen	ER	Reduced cytokine release, reduced Iba1 expression	[53, 54]
Testosterone	AR	Anti-inflammatory response	[55, 56]

Receptors expressed by microglia to peripherally derived hormones. This table includes only hormones discussed in this review and prioritizes hormones that have been studied with microglia-specific receptor manipulation.

### Metabolic-related hormones

Metabolic-related hormones have the potential for large effects on microglia signaling due to microglia expression of receptors for both acute (insulin [46]) and chronic (leptin and adiponectin [48, 50]) energy availability signals. This places microglia as potential integrative nodes in feeding regulation circuits. Insulin has been shown to have an anti-inflammatory effect in microglia, likely through insulin receptor signaling. Insulin is produced by the pancreas in response to increased blood glucose and incretin secretion and acts as a key regulator of fuel utilization throughout the body [58]. In the BV2 cell line, insulin treatment reduced LPS-induced cytokine release and increased phagocytosis [46]. Conversely, in a microglia-specific inducible insulin receptor knock out mouse model, HFD-fed mutant mice had reduced microglia morphology complexity relative to controls, indicating an increase in reactivity [47]. In this study, microglia-specific insulin receptor knock out mice also showed alterations in food intake, suggesting that microglia insulin signaling may directly affect the regulation of feeding behavior.

Insulin has also been investigated for its effect on microglia in multiple neurodegenerative diseases. These include mouse AD models, where intranasal insulin treatment was found to reduce Iba1 and CD68 expression [59, 60]. Although the effect of insulin on microglia in these studies is correlational in nature and thus far lacks a mechanistic explanation, this direction of research is highly translationally significant, as human AD patients have been found to have reduced brain insulin levels, and intranasal insulin administration improved cognitive performance [61, 62]. Understanding insulin’s effect on microglia may help provide a mechanism for this effect. In a mouse PD model, intraperitoneal incretin administration, which leads to insulin production, was found to reduce microglia activation and attenuate dopaminergic cell death [63]. Additional research is needed to uncover the effects of insulin signaling on microglia reactivity in different contexts, as well as to determine the downstream signaling mechanisms through which insulin signals in microglia. It will also be important to understand the effect of chronically high insulin levels seen during the development of insulin resistance and to uncover the mechanisms by which insulin signaling in microglia alters behavior to affect food intake.

Chronic energy availability signals also influence microglia function. These include leptin and adiponectin, which are released by adipose tissue in concentrations that vary in proportion to fat mass. Plasma leptin concentrations increase with increasing fat mass, while adiponectin concentrations decrease [64]. Leptin and adiponectin can have opposing effects on microglia, which express both the leptin receptor (LepR) [48] and adiponectin receptor (AdipoR1 or AdipoR2) [50]. In primary rat microglia cell cultures, leptin treatment enhanced LPS-induced cytokine release [65], while adiponectin treatment prevented LPS-induced cytokine release [50]. Myeloid-specific LepR knock out in mice caused increased body weight, fat mass, and food intake on a standard chow diet [66]. Additionally, hypothalamic microglia were found to have less phagocytic activity, suggesting that leptin signaling enhances microglia phagocytosis, which could in turn affect microglia modulation of neuronal feeding circuits. Studies to date have not challenged microglia-specific LepR knock out mice with HFD, so studying how microglia LepR knockout mice respond to high levels of circulating leptin to affect mouse physiological and microglia response is an important next step. Conversely, pharmacological activation of AdipoR1 and AdipoR2 with AdipoRon, a synthetic, small-molecule adiponectin receptor agonist, promotes microglia quiescence in models of cardiopulmonary bypass surgery [67], chronic unpredictable mild stress [49], and LPS-induced sepsis [68]. It will be important to verify the effect of adiponectin on microglia signaling using perturbations of physiological adiponectin levels in addition to the above pharmacologic manipulations. In addition, AdipoR1 and AdipoR2-specific microglia manipulations will be useful in determining the relative importance of each receptor. Studies thus far suggest that both low leptin and high adiponectin are needed to maintain homeostatic microglia function. They further suggest that adiponectin treatment may be a possible approach to rescue HFD-induced changes in microglia reactivity. Future work is needed to explore this idea and to elucidate what downstream signaling pathways within microglia mediate the pro and anti-inflammatory effects of adipokines on these cells.

### Stress-related hormones

Psychological stress has been shown to influence microglia reactivity and number through corticosterone/glucocorticoid signaling derived from the adrenal cortex, the outer layer of the adrenal glands located on top of each kidney. Microglia responses to stress may differ depending on whether the stress is acute or chronic and the degree and type of the stressor. For example, while glucocorticoid action has immune suppressive effects in the periphery, in microglia glucocorticoid signaling has been found to enhance pro-inflammatory responses [69]. The reasons for this difference are unclear, but microglia-specific glucocorticoid receptor knock out mice showed higher microglia inflammatory gene expression under standard housing conditions. However, when mice were exposed to a chronic mild unpredictable stress paradigm, microglia lacking the glucocorticoid receptor showed reduced inflammatory gene expression [52]. This indicates that microglia glucocorticoid receptor signaling may have an anti-inflammatory effect under basal condition but a pro-inflammatory effect following chronic stress. The mechanisms underlying these differences are not known but may be caused by sensitization of the microglia inflammatory response following a stressful event, resulting in an increased pro-inflammatory response during subsequent stressors, akin to an innate immune memory response [51]. In line with this, chronic corticosterone administration in adrenalectomized rats increased expression of pro-inflammatory microglia markers in the hippocampus [70]. Similarly, in a mouse model of chronically elevated corticosterone levels (db/db mice), pharmacological corticosterone synthesis inhibition reduced microglia number in the hippocampus [71]. It should be noted that both adrenalectomy and the db/db mouse model introduce significant systemic effects, which make it difficult to attribute changes in microglia signaling solely to corticosterone manipulation. The effect of chronic stress on microglia state raises important considerations for preclinical microglia research. These include whether certain housing conditions, such as single animal housing, or animal manipulations, such as injection or drug treatment paradigms, might influence microglia state through glucocorticoid receptor activation. In turn, these responses could significantly influence the treatment being investigated. In designing such studies, control manipulations in animal handling and treatment should be carefully considered to account for microglia responses to stress.

How might microglia responses to stress hormones impact our understanding and treatment of neurological disease? Pharmacological inhibition of mouse microglia activation with minocycline treatment was shown to prevent some of the depressive-like behavior seen with chronic stress by improving social exploration and sucrose preference [72]. While not specific to microglia, post-mortem human brain data supports the idea that elevated inflammatory and stress markers are associated with bipolar disorder and schizophrenia [73], and anti-inflammatory medication can be an effective treatment in some cases of treatment resistant schizophrenia [74]. Chronic stress may also impact microglia interactions with neurons through decreased trophic support and impaired Aβ clearance, contributing to neurodegenerative pathology [75, 76]. Indeed, it has been suggested that stress-induced changes in microglia function exacerbate AD pathology, although more studies are needed to determine the exact mechanisms responsible [77]. At the translational level, stress reduction is known to decrease the risk for diseases like dementia, which could in part reflect a role for chronic stress in increasing microglia inflammatory responses [78].

### Reproductive fitness hormones

Microglia can also respond to cues related to reproductive fitness and are sensitive to gonadal steroid hormones, such as estrogen and testosterone, through their expression of both the estrogen receptor [54] and androgen receptor [55]. Notably, both classes of receptors have been shown to reduce pro-inflammatory responses in microglia. RNA sequencing of isolated microglia from healthy adult mice show transcriptional differences between male and female microglia, with male microglia having significantly higher expression of pro-inflammatory genes such as NF-κB [79]. At a broader level, microglia number has been found to vary by region in a sex-dependent manner, with male mice having greater Iba1 density in the hippocampus, cortex, and amygdala in adulthood [80]. Sex-specific difference in microglia morphology have been found as early as embryonic day 18, with specific regions in both male and female mice showing greater ameboid microglia at specific developmental timepoints [81]. Sex-specific differences in microglia function and responses to external stimuli has been reviewed extensively [81], and these studies highlight the importance of sex on microglia development and reactivity. Here, we instead focus on how direct administration of reproductive hormones can influence microglia function and disease outcomes.

Reproductive fitness hormones can influence neurological disease. Male mice with traumatic brain injury (TBI) treated with estradiol, the most potent form of estrogen, had reduced neuronal death and improved neurobehavioral outcomes. They also showed reduced Iba1, TLR4 and NF-κB expression, as well as reduced expression of pro-inflammatory cytokines such as IL-6, TNF-α, and IL-1β [53]. Estradiol treatment was also found to suppress Nlrp3 induction and IL-18 expression in female mice in a multiple sclerosis model, and improved behavioral outcomes and myelination levels [82]. In a related study, estradiol also prevented HFD-induced changes in microglia morphology in the arcuate nucleus of ovariectomized rats [83]. Because these studies use systemic estradiol treatment, further research using microglia estrogen receptor knock out models to assess if the anti-inflammatory effects persist will help establish a cell-specific basis for estrogen’s effect on microglia. The effect of testosterone on microglia has been less studied. However, it has been shown that microglia androgen receptor expression is upregulated in a demyelinating model, and myeloid-specific androgen receptor knockout mice show reduced in testosterone-induced anti-inflammatory responses [56]. Androgen receptor expression is also enhanced in microglia after TBI [55], and testosterone administration reduces the amount of reactive microglia around the injury site, although this study did not report how these changes affected injury progression [84]. Microglia response to estrogen and testosterone underlines the importance of using both sexes in preclinical research.

Finally, emerging research suggests that microglia may be responsive to many more hormones, such as thyroid hormone [85] and renin [86], as well as brain-derived hormones such as melatonin [87]. The complex response of microglia to hormones means that any experimental manipulation that modifies multiple hormones, such as dietary changes, stress models, and circadian manipulations will require careful dissection to understand what changes in microglia state are due directly to the manipulation and what changes are due to potential secondary changes in hormones.

## Gut microbiome

The gut microbiome can modulate microglia function through three avenues—vagus nerve signaling, bacterial metabolites, and modulation of intestinal permeability (reviewed in Madore, Yin et al. [76]). Vagus nerve stimulation in mice has been found to have a protective effect on microglia in multiple disease contexts that suggest improved disease outcomes, including in AD [88], Parkinson’s disease (PD) [89], LPS-induced inflammation [90], and cerebral ischemia/reperfusion injury [91, 92]. However, the causal mechanism by which vagus nerve stimulation modulates microglia state is not well understood and likely has brainstem signaling as an intermediate. Thus, we focus on other facets of the microbiome here.

Modulation of the microbiome is often accomplished using germ-free (GF) mice, antibiotic treatment, or dietary manipulation, each of which induces off target effects. Consequently, it can be difficult to determine direct causality of changes in microglia function. With this caveat in mind, both the effect of bacterial metabolites, especially short chain fatty acids (SCFAs), and intestinal permeability through LPS treatment have been studied in microglia. Microbiome alteration, such as with chronic antibiotic treatment, has been shown to have protective effects in mouse models of AD [93] and PD [94], although antibiotic treatment had no effect in a mouse multiple sclerosis (MS) model [95]. Conversely, microbiome alteration via fecal microbiota transfer did improve outcomes in a mouse MS model [96]. Thus, manipulating microbiome may have translational potential for some neurodegenerative diseases. Here, we highlight specific microbe and microbiome produced signals to which microglia can directly respond.

### Short chain fatty acids

Among the many metabolites produced by gut bacteria, SCFAs have received perhaps the most attention [97]. SCFAs can cross the BBB through monocarboxylate transporters (MCTs). There are three main types of SCFAs, acetate, propionate, and butyrate, which are primarily produced during bacterial fermentation of dietary fiber [98]. Germ-free mice that lack a microbiome were found to have altered microglia reactivity and morphology across brain regions, including the cortex, hippocampus, and cerebellum, which was normalized with SCFA supplementation [99]. Colonization of GF mice with three defined strains of bacteria, as opposed to the hundreds normally present, was not sufficient to promote normal microglia morphology, suggesting that a diversity of bacteria strains is required [99]. A follow up study by Erny, Dokalis et al. [100] investigated how SCFAs impact microglia function and found that microglia metabolic pathways were altered in GF mice, and that these alterations were normalized following supplementation specifically with the SCFA acetate. In line with these findings, mice fed a fiber-deficient diet showed increased microglia reactivity and increased microglia phagocytosis of synapses in the hippocampus, which was restored with SCFA supplementation [101]. Additional studies examining the effect of SCFAs on microglia in conventional as opposed to GF mice, as well as in mice fed a standard diet, will help determine the effect of SCFA supplementation under homeostatic conditions.

The effect of SCFA supplementation on microglia in disease models has not been well studied, though there are some results that hint at important roles in disease. For example, one study found that SCFA supplementation reduced microglia number and reactivity in the mouse cortex following stroke, which was associated with improved behavioral outcomes and cortical plasticity [102]. Conversely, SCFA supplementation promoted microglia activation in a mouse model of PD and worsened motor dysfunction [103]. These contrasting results highlight the importance of examining microglia response to SCFA in different disease contexts and duration and supplementation regiments, such as acute vs chronic administration. In humans, individuals with neurodegenerative diseases, such as AD [76] and PD [104], display microbiome alterations and reduced SCFA production, which suggests that altered SCFA levels have the potential to play a role in microglia alterations observed in those conditions. These changes are mirrored in mouse models of these diseases [105, 106]. Since SCFA administration is not specific to microglia, assessing what effects are directly due to SCFA action on microglia will be an important area of research for future studies. Toward this goal, microglia-specific knockout of SCFA transporters such as FFARs or MCTs could be investigated, and direct brain administration of a physiological dose of SCFAs would help minimize off target effects.

### Amino acid metabolites

Fermentation of dietary amino acids by gut microbiota leads to the production of bioactive amino acid metabolites, which can also signal to microglia. One particular class of metabolites that has been studied in microglia signaling are indoles, which are derived from microbiota tryptophan metabolism and act on the aryl hydrocarbon receptor (AhR) [107]. Microglia AhR activation has been primarily examined in disease contexts, although one study examining healthy mice found that global AhR knockout attenuated the pro-inflammatory effects of LPS treatment in cortical microglia [108]. Similarly, in an APP/PS1 AD mouse model, indole supplementation in mice fed a tryptophan deficient diet reduced cortical microglia Iba1 levels, inflammatory cytokine levels, and NF-κB pathway activation, which was associated with reduced Aβ accumulation and improved cognitive function [109]. This same effect was seen in a mouse sepsis model, where oral gavage of indole reduced cortical microglia Iba1 levels after sepsis induction. Moreover, the survival benefit of indole supplementation in sepsis was dependent on microglia, as revealed by microglia depletion [110]. Studies assessing how indole supplementation affects microglia signaling outcomes with a standard diet and under homeostatic conditions will be critical to understanding the translational potential of indole supplementation. Depending on the timing and progression of the disease, less reactive microglia may be deleterious to disease progression, and it has also been suggested that microglia AhR activation may promote reduced amyloid beta plaque clearance in AD [111]. The role of bacteria metabolites on microglia state is a relatively new direction of study, and further research examining chronic effects of metabolite modulation in both healthy and disease contexts will be necessary to understand the mechanism by which the gut microbiota contributes to microglia function. In this context, microglia specific manipulations of relevant receptors will again be an important approach.

### Lipopolysaccharide

LPS is a Gram-negative bacteria component that acts as a potent TLR4 agonist. High levels of LPS are present in the blood stream in sepsis [112]. In the context of increased in gut permeability, LPS levels in the bloodstream increase as well but to a lesser extent than in sepsis, resulting in metabolic endotoxemia and chronic low-grade inflammation [113]. Increased plasma LPS concentrations are also associated with conditions such as diabetes and obesity [114] and several neurological conditions, including AD [115], PD [116], and MS [117]. In addition, elevated LPS levels have been found in the hippocampus and cortex of human AD patients [118]. Thus, understanding the role of LPS in neurological disease progression is an important objective. Toward this goal, LPS has been shown to enter the brain at circumventricular organs and ventricles [119], raising the possibility that LPS may affect microglia state in regions such as the arcuate nucleus and hippocampus. LPS has been extensively used both in vitro and in vivo to induce and study pro-inflammatory microglia responses [120, 121]. Although LPS is used in this way as a tool to induce pro-inflammatory microglia responses, in the context of modeling disease with LPS, the concentration and route of LPS delivery needs to be carefully considered. I.V. administration of 1 ng/kg LPS in humans was sufficient to induce microglia activation and a physiological sickness response [122], while mice may require a significantly larger dose to induce the same response (e.g. 2 mg/kg [123]) due to reduced sensitivity. Studies examining the effect of gut-originating LPS on microglia are limited, and understanding the mechanisms by which gut-derived LPS modulates microglia state in vivo is an important future direction of study.

## Conclusion

A growing body of evidence demonstrates that peripheral cues—ranging from systemic inflammation and circulating cytokines to metabolic signals and microbial metabolites—exert profound influence on microglial phenotype and function. These peripheral signals can cross or interact with the blood-brain barrier through various mechanisms, including passive diffusion, transport of soluble mediators, and vagal afferents, thereby shaping microglial responses in both homeostatic and pathological contexts. Such modulation affects critical microglial roles in the orchestration of neuroimmune responses such as cytokine release, potentially affecting synaptic pruning and neural engulfment, which in turn could impact both neural development and neural disease progression. This highlights the importance of an integrative, systems-level approach to studies of microglia that takes into account the bidirectional communication between the CNS and peripheral physiological states in the body. These considerations are especially important in understanding human health given that 95% of Americans do not meet the recommended fiber intake and that 42% of Americans are obese [124].

Several critical questions remain unanswered regarding the precise mechanisms and contextual factors that govern peripheral regulation of microglial function. For example, it will be important to explore the relative contribution of microglia-specific receptor responses compared to secondary peripheral influences on microglia state. To test this, available microglia-specific knockouts, such as the Tmem119-creER mouse, are critical [125]. An additional consideration is the importance of using an inducible cre to avoid developmental compensation. It will also be crucial to resolve the mechanisms by which peripheral cues interact to influence microglia state and to determine how these cues interface with each other when multiple signals are present. The studies presented in this review have examined factors in isolation but given that many of these cues impinge on conserved downstream pathways (e.g. inflammatory responses), they are likely to have combinatorial effects. Given the role for sex-specific differences and stress-induced responses in microglia outcomes, another important area of study is how physiological context impacts microglia responses to peripheral cues. Most studies have examined microglia responses in a limited experimental paradigm, such as in healthy mice, young mice, male mice, or in limited treatment dosages or durations. Understanding how microglia responses change in acute vs chronic treatment, in female mice, in different brain regions, and in different disease models with these variables will improve our understanding of the role of microglia in brain regulation across contexts. Finally, microglia function and response properties can vary across brain regions, in different microglia states, and with different durations downstream of innate immune priming or tolerization responses. It is presently unknown whether all microglia are equally responsive to the peripheral cues discussed here and how different conditions or disease state may influence short- and long-term changes in the ability of microglia to recognize and respond to these cues. Research to answer these and related questions will not only advance our understanding of microglial plasticity but may also present novel peripheral targets that are more readily assessable, such as diet or environment, for therapeutic intervention in neurodegenerative disease.

## Data Availability

Not applicable.

## Abbreviations

Aβ: Amyloid beta
AD: Alzheimer’s disease
AdipoR1/2: adiponectin receptors 1 and 2
AhR: Aryl hydrocarbon receptor
AR: Androgen receptor
BBB: Blood brain barrier
CSF: Cerebral spinal fluid
ER: Estrogen receptor
FFAR4: Free fatty acid receptor 4, also known as GPR120
GF: Germ-free
GR: Glucocorticoid receptor
HFD: High fat diet
InsR: Insulin receptor
LepR: Leptin receptor
LPS: Lipopolysaccharide
MCT: Monocarboxylate transporter
MIND: Mediterranean-DASH Intervention for Neurodegenerative Delay
MS: Multiple sclerosis
PD: Parkinson’s disease
PUFA: Polyunsaturated fatty acid
SCFA: Short chain fatty acid
SVCT2: Sodium-dependent vitamin C transporter 2
SFA: Saturated fatty acid
TBI: Traumatic brain injury
TLR4: Toll-like receptor 4
VDR: Vitamin D receptor
